# Sustainable and Efficacy Approach of Green Synthesized Cobalt Oxide (Co_3_O_4_) Nanoparticles and Evaluation of Their Cytotoxicity Activity on Cancerous Cells

**DOI:** 10.3390/molecules27238163

**Published:** 2022-11-23

**Authors:** Noha Al-Qasmi

**Affiliations:** Chemistry Department, Faculty of Science, Taif University, Al-Hawiah, Taif City P.O. Box 11099, Saudi Arabia; n.algassimy@gmail.com or noha.alqasmi@tu.edu.sa

**Keywords:** green approach, Co_3_O_4_ nanoparticles, rosemary leaf extract, liver cancer, cytotoxicity

## Abstract

In this study, rosemary leaf extract was effectively used to synthesize cobalt oxide nanoparticles (Co_3_O_4_ NPs) using a rapid, low-cost, and environmentally friendly approach. The prepared Co_3_O_4_ NPs were examined using various analytical techniques. However, UV spectrum analysis displayed two sharp absorption peaks at ~350 and 745 nm. The dynamic light scattering and zeta potential measurements were used to evaluate the particle size and the effective stabilization of the synthetic nanoparticles in the suspensions. A semi-triangular pyramidal shape of the Co_3_O_4_ NPs with a wide particle-size distribution could be observed in the scanning electron microscopy images. The energy-dispersive X-ray spectrum confirmed their successful synthesis, as the experimental atomic percentages agreed with the theoretical values. Moreover, X-ray diffraction analysis revealed that the synthesized Co_3_O_4_ NPs had a cubic crystalline structure corroborating that of theoretical Co_3_O_4_. Additionally, the Co_3_O_4_ NPs were not toxic at ≤62.5 µg/mL for Hep G2 and at ≤31.25 µg/mL for Mcf7. Therefore, these unique environmentally friendly Co_3_O_4_ NPs at this safe concentration could be studied in the future for their therapeutic activity.

## 1. Introduction

Extensive research on nanostructured materials has provided access to new classes of functional materials with novel characteristics and uses. Owing to their large surface areas, unusual adsorptive characteristics, and rapid diffusivities, nanosized crystalline metal oxides have attracted increasing attention in recent years. P-type semiconductor cobalt oxide nanoparticles (Co_3_O_4_ NPs) are transition metal oxides that can exist in various oxidation states, including Co^2+^, Co^3+^, and Co^4+^ [1,2,3,4]. Among them, the spinel structure is the most stable form of Co_3_O_4_ NPs, which can be used in a wide range of applications, including lithium-ion batteries, gas sensors, supercapacitors, solar selective absorbers, drug delivery, anticancer and antimicrobial agents, water remediation, and photocatalysis [2,5,6,7,8,9,10]. Additionally, the outstanding magnetic, optical, chemical, physical, thermal, and biomedical characteristics of Co_3_O_4_ NPs are of particular interest. Further, Co_3_O_4_ NPs are more economical metal oxide nanoparticles than other noble metals [11,12]. Co_3_O_4_ NPs had previously been investigated as therapeutic agents for treating disorders such as microbial infections, rendering them attractive for use in biomedical applications [13]. In comparison to antibiotics, Co_3_O_4_ NPs have fewer side effects, and higher antibacterial and antifungal properties at low concentrations, and are nontoxic to the body at low levels [14].

Coprecipitation, sonochemistry, chemical spray pyrolysis, thermal decomposition, solution combustion, microwave-assisted, microemulsion, and hydrothermal reactions are examples of physical and chemical processes that have been used to synthesize Co_3_O_4_ NPs [4,15,16,17]. The abovementioned synthesis techniques, although effective, have a number of disadvantages, such as being expensive, time-consuming, energy-intensive, and harmful to the environment [5]. The use of plants, plant components, and microorganisms (algae, bacteria, and fungi) that can produce NPs is a more environmentally friendly approach that meets the demands of minimal hazardous waste [2]. Various studies have reported the synthesis of Co_3_O_4_ NPs using plants such as *Calotropis gigantea*, *Moringa oleifera*, *Aspalathus linearis*, *Terminalia chebula*, *Sageretia thea*, *Calotropis procera*, *Manihot esculenta* Crantz, and *Euphorbia heterophylla* L. [18,19,20,21,22,23].

**Figure 1:**
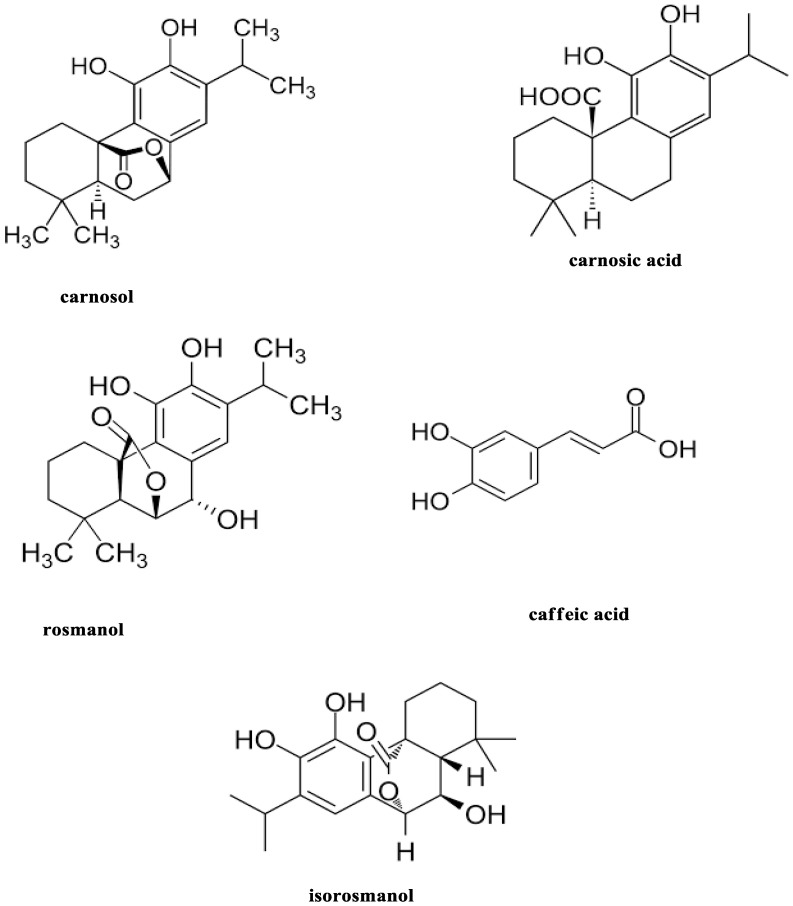
Chemical structure of biologically active phytochemicals in rosemary leaves.

Rosemary (*Rosmarinus officinalis* Linn.), a common household plant, an evergreen perennial aromatic shrub native to the northern and southern coasts of the Mediterranean Sea [24]. Recent research has suggested that traditional medicinal herbs may help in preventing or treating certain metabolic problems, including diabetes, heart disease, and some types of cancer [25]. Dried rosemary leaves and flowers are particularly interesting sources of biologically active phytochemicals because they contain a number of phenolic compounds with significant in vitro antioxidant activity, such as carnosol, carnosic acid, rosmanol, 7-methyl-epirosmanol, isorosmanol, rosmadial, and caffeic acid [26,27], as shown in Figure 1.

Using rosemary leaf extract as a capping and reducing agent, Co_3_O_4_ NPs were synthesized using a completely green approach wherein no organic or inorganic solvents, surfactants, or other chemicals were used. The characterization and cytotoxic activity of the Co_3_O_4_ NPs on liver and breast cancer cells are also described in this study.

## 2. Results and Discussion

### 2.1. UV Spectroscopy

**Figure 2:**
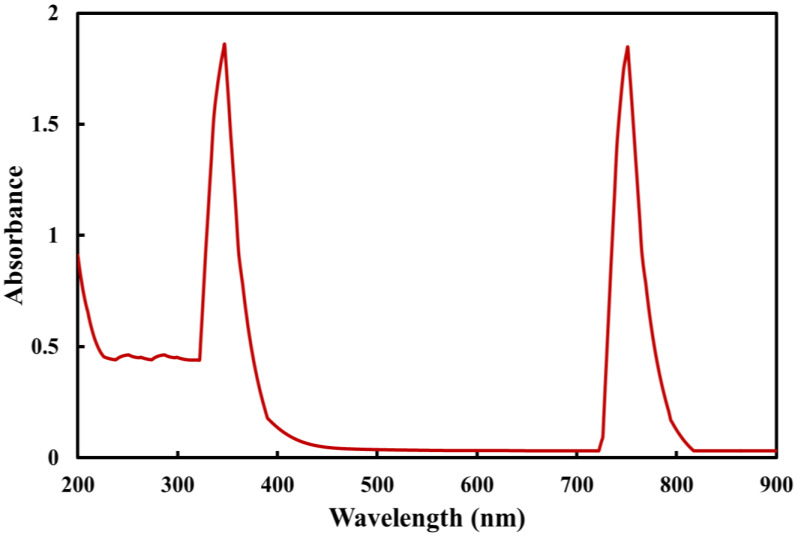
UV spectrum of Co_3_O_4_ NPs synthesized using rosemary leaf extract.

A color change into dark brown was initially used to identify the green synthesis of Co_3_O_4_ NPs. The chemical process between the components of rosemary leaf extract and metal ions was examined using the UV–visible spectrum. The UV spectrum of Co_3_O_4_ NPs synthesized using rosemary leaves indicated nanoparticle formation by demonstrating two sharp absorption peaks at approximately 350 and 745 nm (Figure 2). However, these two different wavelengths might have arisen from the charge transfer process from the ligand to the metal as O_2_^−^ → Co^2+^ for 350 nm absorption peak, and O_2_^−^ → Co^3+^ for the 745 nm absorption peak. In addition, these peaks were attributed to surface plasmon resonance behavior [28].

### 2.2. Dynamic Light Scattering (DLS) and Zeta Potential

**Figure 3:**
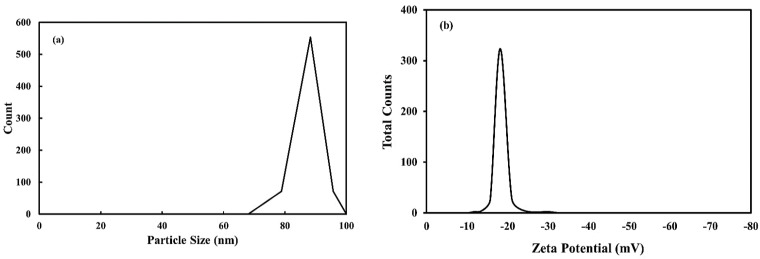
(**a**) DLS measurements of particle size distribution and (**b**) charge zeta potential of Co_3_O_4_ NPs synthesized using rosemary leaf extract.

The particle size distribution for the Co_3_O_4_ NPs synthesized using rosemary leaf extract, as determined by the DLS method, is displayed in Figure 3a. The average particle size distribution of the synthesized Co_3_O_4_ NPs was 89 nm depending on size distribution data. Analysis revealed a unimodal size distribution with polydispersity indices; the suspension was monodispersed and produced high colloidal stability. Moreover, the zeta potential of the synthesized Co_3_O_4_ NPs showed a peak at −18 mV (Figure 3b). This result reveals that the surface of the synthesized nanoparticles acquired a negative charge, and the nanoparticles dispersed significantly throughout the medium. Therefore, the determined negative value was responsible for the good stabilization of prepared NPs in the suspensions. Furthermore, because the average size is a measure of hydrodynamic size, its value indicates both the availability of nanoparticles and any solvent molecules linked to the tumbling particle.

### 2.3. TEM

**Figure 4:**
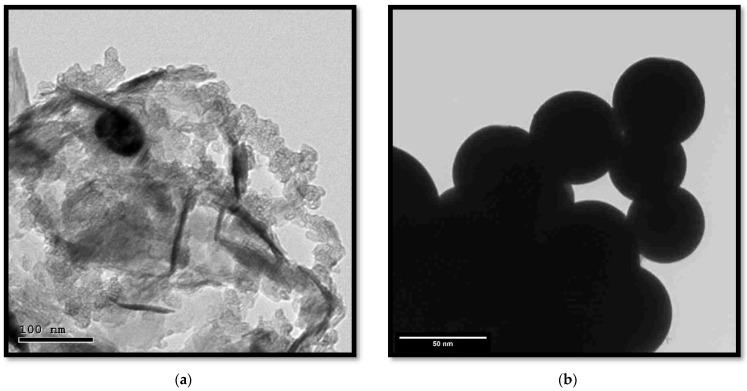
(**a**) Transmission electron microscopy image and (**b**) high-resolution transmission electron microscopy image of the Co_3_O_4_ NPs synthesized using rosemary leaf extract.

The microstructure of the synthesized Co_3_O_4_ NPs using rosemary leaf extract was investigated using the TEM and HRTEM images shown in Figure 4a,b. As depicted in Figure 4a, the prepared nanoparticles had an almost irregular cluster shape, with the presence of some rod-like and sub-spherical regions with a diameter of approximately 100 nm. The HRTEM image (Figure 4b) reveals agglomerated spherical nanoparticles. The average particle size was in the approximate range of 50–100 nm.

### 2.4. SEM

**Figure 5:**
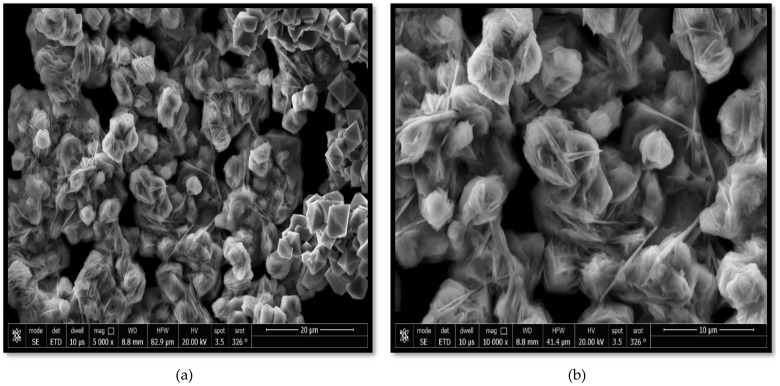
(**a**,**b**) Scanning electron microscopy images of Co_3_O_4_ NPs synthesized using rosemary leaf extract at different magnifications.

The morphologies of the Co_3_O_4_ NPs synthesized using rosemary leaf extract at various magnifications are shown in Figure 5a,b, where a semi-triangular pyramidal shape with various-sized particles is shown. The magnetic induction characteristics of Co_3_O_4_ NPs may have been the reason for the highly agglomerated synthesized nanoparticles [1]. This result is consistent with that of TEM studies.

### 2.5. EDX Spectrum

**Figure 6:**
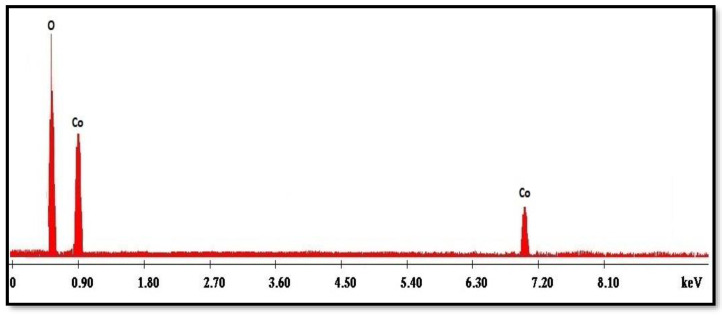
Energy-dispersive X-ray spectrum of the Co_3_O_4_ NPs synthesized using rosemary leaf extract.

Figure 6 displays the EDX spectrum of the Co_3_O_4_ NPs synthesized using the rosemary leaf extract. The major components of the synthesized nanoparticles were only O and Co, with atomic percentages of 24 and 75.9%, respectively; this is almost identical to the theoretical 3:4 ratio in Co_3_O_4_ NPs [14].

### 2.6. Crystalline Structure

**Figure 7:**
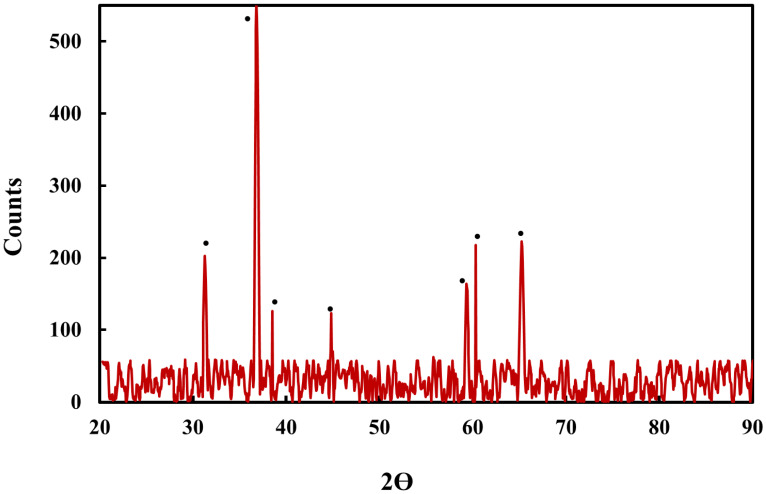
X-ray diffraction pattern of Co_3_O_4_ NPs synthesized using rosemary leaf extract.

The XRD pattern of the Co_3_O_4_ NPs synthesized using rosemary leaf extract is depicted in Figure 7, which shows that the NPs had a cubic phase structure. The peak positions (2θ = 31.27°, 36.85°, 38.55°, 44.82°, 59.37°, 60.37°, and 65.25°) and relative intensities of the Co_3_O_4_ NPs matched those of the JCPDS card no. COD 9005887 file, confirming Co_3_O_4_ with a cubic structure Fd-3m (227) space group. However, no typically observed impurity peaks [29] were observed. Using the Scherrer relation, the average crystalline size was approximately 90 nm.

### 2.7. Cytotoxicity Activity

The in vitro cytotoxicity evaluation of synthesized Co_3_O_4_ NPs is crucial for biomedical applications. To determine the optimal concentration where there is no cytotoxic effect on living tissue, and to demonstrate the safety of synthesized NPs for further research, different concentrations of the synthesized Co_3_O_4_ NPs using rosemary leaf extract were tested against Hep G2 and Mcf7 cancer cell lines.

As the liver is the primary site for chemical and food metabolism, liver carcinoma cells were used. Regardless of the method of administration (intravenous injection or orally via the digestive tract to reach the blood circulation system), the drug must pass through the liver. In addition, many chemicals undergo a metabolic process in the liver before elimination, leading to the possibility of liver poisoning. Therefore, the liver is a major organ that must be protected when receiving chemicals. Hepatic cell cancer is the fourth most common cause of cancer-related death. Additionally, because of its distinct structure and function, the liver plays a significant role in the immune system and inflammation [30,31].

**Figure 8:**
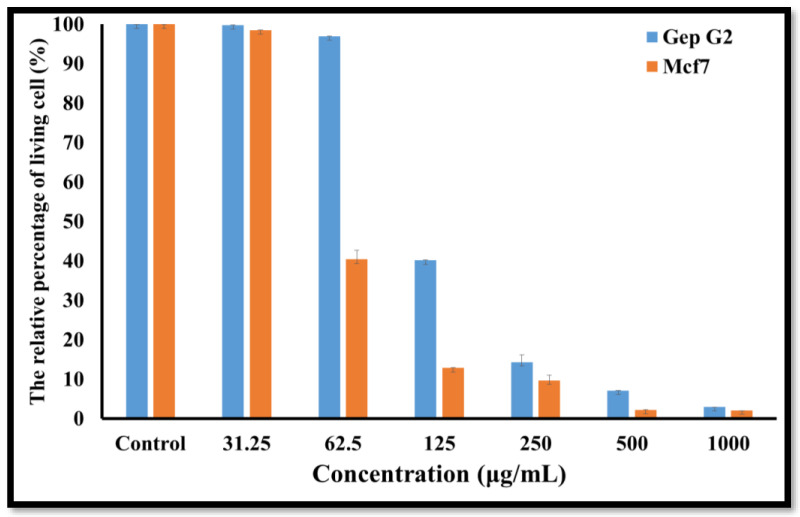
Relative percentages of living cells of Hep G2 and Mcf7 cancer cells after exposure to different concentrations of Co_3_O_4_ NPs synthesized using rosemary leaf extract. The results are from an MTT assay and are displayed as the mean ± SD (n = 3).

**Figure 9:**
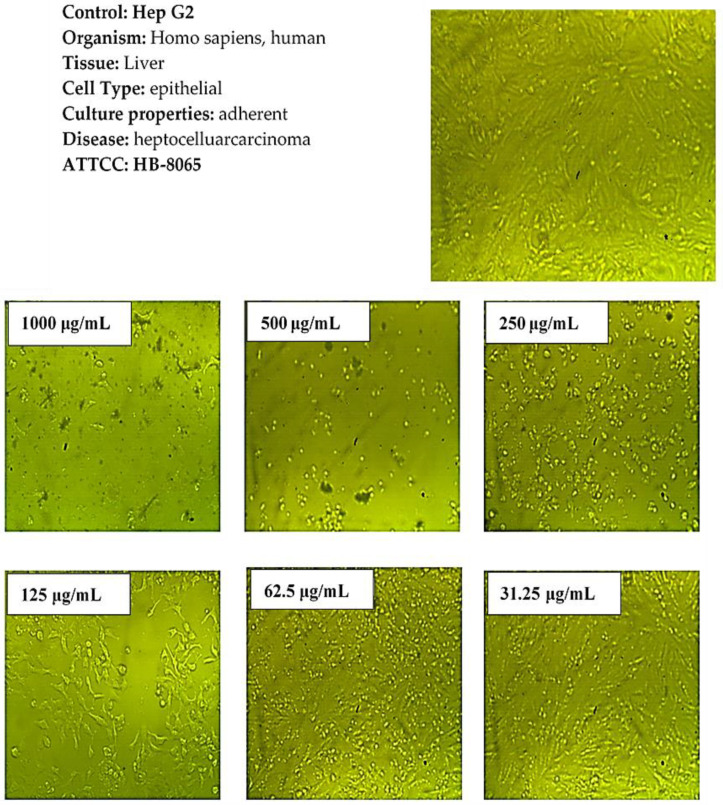
Microscopic images of Hep G2 cancer cells after treatment with various concentrations of Co_3_O_4_ NPs synthesized using rosemary leaf extract.

**Figure 10:**
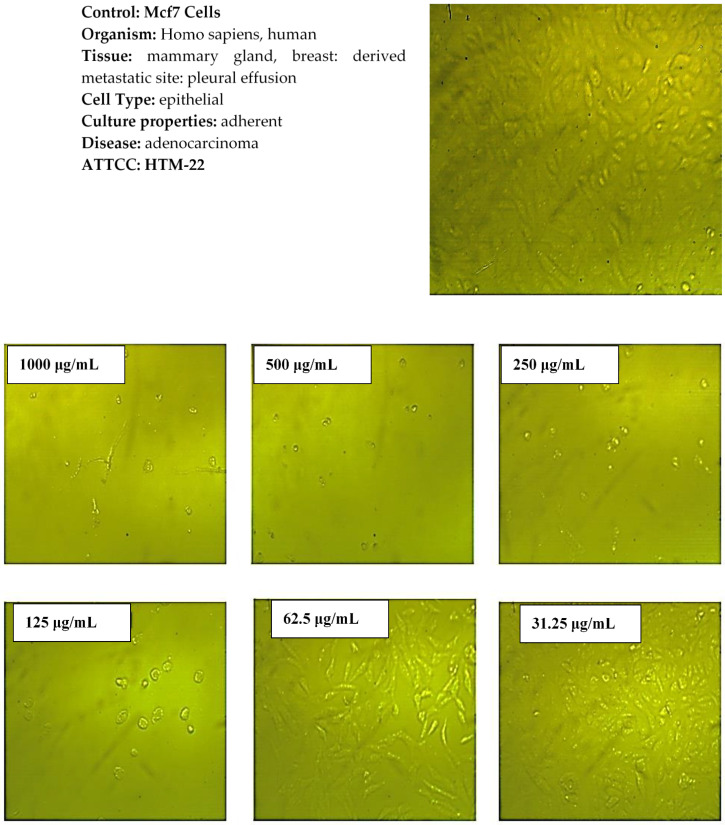
Microscopic images of Mcf7 cancer cells after treatment with various concentrations of Co_3_O_4_ NPs synthesized using rosemary leaf extract.

Figure 8 demonstrates the effect of the synthesized Co_3_O_4_ NPs using rosemary leaf extract on the metabolic activity of the Hep G2 and Mcf7 cancer cell lines following a 24 h incubation time at various concentrations (31.25, 62.5, 125, 250, 500, and 1000 μg/mL) by using MTT assay. The results show that the metabolism of the Hep G2 and Mcf7 cell lines, when compared to the positive control, was not affected by the concentration of Co_3_O_4_ NPs ≤62.5 µg/mL for Hep G2 and ≤31.25 µg/mL for Mcf7. This indicates that Co_3_O_4_ NPs are not toxic up to ≤62.5 µg/mL for Hep G2, and ≤31.25 µg/mL for Mcf7, which was also observed from microscopic images of Hep G2 and Mcf7 after applying those lower concentrations. The examined nanoparticles had a noticeable effect on the cell viability of both cancerous cell lines at higher concentrations. As illustrated in Figure 8, when the concentration of synthesized Co_3_O_4_ NPs was increased to 125 μg/mL, the metabolic activity of the Hep G2 and Mcf7 cancer cell lines decreased. This indicates that Co_3_O_4_ NPs had a toxic effect on Hep G2 and Mcf7, which was observed more when higher concentrations were applied. The microscopic images of the Hep G2 and Mcf7 cancer cell lines that were exposed to various doses of the synthesized Co_3_O_4_ NPs using rosemary leaf extract support these outcomes (Figure 9 and Figure 10). The half-maximal inhibitory concentrations values (IC_50_) were also determined to be 112 μg/mL for Hep G2 and 86 μg/mL for Mcf7. These in vitro findings indicate that the synthesized Co_3_O_4_ NPs are safe to be used in concentrations ≤ 31.25 µg/mL.

## 3. Experiments

### 3.1. Materials

Cobalt chloride hexahydrate (CoCl_2_·6H_2_O) was purchased from Sigma-Aldrich (St. Louis, MI, USA) and used as received. Double-distilled water was used in this study.

### 3.2. Preparation of the Rosemary Leaf Extract

The rosemary was purchased from a local market and rinsed with tap water. Subsequently, it was cleaned with double-distilled water to eliminate any residues and then air-dried for six days. Approximately 5 g of dried leaf was combined with 100 mL of double-distilled water to create the extract, which was then heated for 20 min at 50 °C. The extract was then filtered and stored for further research [32].

### 3.3. Biosynthesis of the Co_3_O_4_ NPs

In a flask, 100 mL of double-distilled water was used to dissolve 10 g of CoCl_2_·6H_2_O. The solution was heated to 50 °C and kept at this temperature for 5 min on a hot plate. The salt solution was then supplemented with 20 mL of rosemary extract while being vigorously stirred. During the reaction, the solution turned dark brown, indicating the synthesis of Co_3_O_4_ NPs. Following this, the mixture was centrifuged for 20 min at 15,000 rpm. The synthesized Co_3_O_4_ NPs were subsequently rinsed with double-distilled water and dried for 24 h at 80 °C. Subsequently, the dried Co_3_O_4_ NPs were annealed at 600 °C for 2 h in a muffle furnace [14] for stability. The synthesized Co_3_O_4_ NPs were then placed in a glass vial and stored for characterization.

### 3.4. Characterization of the Co_3_O_4_ NPs

UV, VIS, and NIR spectrometers were used to investigate the synthesized Co_3_O_4_ NPs spectrum (Lambda 750, Parkin Elmer). Furthermore, the surface charge and particle size of the prepared Co_3_O_4_ NPs were determined using a zeta seizer instrument (NanoSight NS500, Malvern Panalytical, Malvern, UK). The morphology and elemental composition of the produced Co_3_O_4_ NPs were investigated using energy-dispersive X-ray spectroscopy (EDX) analysis and scanning electron microscopy (SEM; JSM-6701FPlus, JEOL Ltd., Tokyo, Japan) at an acceleration voltage of 18 kV and magnifications of 5000× and 30,000×. Transmission electron microscopy (TEM) was performed using a high-resolution JEM-2100 instrument (JEOL Ltd.). The X-ray diffraction (XRD; EQUINOX 1000, Thermo Fisher Scientific, Waltham, MA, USA) patterns of powdered Co_3_O_4_ NPs were obtained using Cu–Kα radiation with a current of 35 mA and an applied voltage of 39 kV. The 2θ angles ranged from 20° to 90° with a scan speed of 0.3°/min.

### 3.5. In Vitro Cytotoxicity Evaluation

An MTT assay was used to evaluate the in vitro cytotoxicity of the synthesized Co_3_O_4_ NPs against liver cancer cells (Hep G2, ATCC number HB-8065) and cancerous breast cells (Mcf7, ATCC number HTB-22). To fabricate a complete monolayer sheet, a 96-well tissue culture plate was incubated with 1 × 10^5^ cells/mL (100 µL/well) at 37 °C for 24 h. After the formation of a confluent sheet, the growth medium was isolated from the 96-well microtiter plates, and the cell monolayer was washed twice with the wash medium. The analyzed samples were diluted twice in an RPMI-1640 medium containing 2% serum (maintenance medium). Three wells served as the control and received only the maintenance medium, while 0.1 mL of each dilution was tested in different wells. After testing, the plates were incubated at 37 °C. Physical characteristics of toxicity were examined in the cells, such as the complete or partial loss of the monolayer, rounding, shrinkage, and cell granulation. An MTT solution (5 mg/mL in PBS) was prepared (Bio Basic Inc., Markham, ON, Canada), and 20 µL of the solution was added to each well. To completely blend MTT into the medium, the samples were shaken at 150 rpm for 5 min. The samples were then incubated for 4 h at 37 °C with 5% CO_2_ to allow for MTT to be metabolized. The medium was then removed, and the plates were dried on paper towels to remove any residues. To thoroughly mix the formazan and solvent, 200 µL of DMSO was used to resuspend the formazan (MTT metabolic products) before shaking at 150 rpm for 5 min. The background was subtracted at 620 nm before calculating the optical density at 560 nm. The cell number and optical density were directly correlated.

## 4. Conclusions

Co_3_O_4_ NPs were prepared with a practical ecofriendly approach using an aqueous extract of rosemary leaves. Different analytical methods were used to analyze the synthesized Co_3_O_4_ NPs. The UV spectrum revealed two distinct sharp absorption peaks that confirmed the successful synthesis of Co_3_O_4_ NPs using the rosemary leaf extract. The excellent stability of synthesized nanoparticles in the suspensions and the particle size were evaluated using studies of dynamic light scattering and zeta potential. SEM images showed that the synthesized Co_3_O_4_ NPs had a semi-triangular pyramidal form with wide particle-size distribution, while EDX analysis confirmed their successful synthesis. XRD analysis confirmed the crystal structure of the prepared NPs. Furthermore, the synthesized Co_3_O_4_ NPs were not toxic to Hep G2 (at ≤62.5 µg/mL) and Mcf7 (at ≤31.25 µg/mL), so this novel ecofriendly Co_3_O_4_ NPs at this safe concentration can be evaluated in the future for its therapeutic activity.

## Data Availability

Data are contained within the article.
